# Vaccination coverage and hesitancy in Brazil: survey reveals reality and offers inputs for the National Immunization Policy

**DOI:** 10.1590/S2237-96222024v33e2024638.especial2.en

**Published:** 2025-01-10

**Authors:** Eder Gatti Fernandes, Jadher Percio, Ethel Leonor Noia Maciel

**Affiliations:** 1 Ministério da Saúde, Secretaria de Vigilância em Saúde e Ambiente, Brasília, DF, Brazil

## INTRODUCTION

Vaccination coverage in Brazil increased significantly with the implementation of the National Health System (*Sistema* Único *de Saúde –* SUS) in the 1990s, being above 95% for several vaccines and contributing to the control and elimination of several vaccine-preventable diseases.^1^ However, the National Immunization Program (*Programa Nacional de Imunizações* - PNI), in operation since 1973, currently faces obstacles, such as vaccine hesitancy, driven by misinformation, low risk perception and geographic and administrative barriers in accessing vaccination services.^2^

Between 2020 and 2021, a national survey, coordinated by the Faculty of Medical Sciences of the *Santa Casa de São Paulo*, was conducted in all 26 state capitals and the Federal District, in partnership with the PNI.^3^ The survey investigated vaccination coverage of children born between 2017 and 2018, assessed access to vaccination services, compared data from vaccination cards with administrative records and identified causes of vaccine hesitancy. With a sample of 37,836 children, stratified by socioeconomic level and using sampling weights, the survey faced challenges during the COVID-19 pandemic, especially due to physical distancing restrictions imposed by municipal governments.

The results of the survey help to understand the current challenges faced by the PNI. The survey found that no vaccine provided for in the National Vaccination Schedule for children up to 2 years of age achieved 95% vaccination coverage, with most of the estimated parameters being between 80% and 90%. In several capitals, even in higher socioeconomic strata, coverage did not reach targets, highlighting regional and social inequalities. Approximately 30% of children received at least one vaccine in private services, whereby this proportion was higher among families in the highest socioeconomic stratum (58.9%), when compared to the lowest stratum (6.1%).

Confidence in public service vaccines was high across all strata, with more than 90% of those responsible for the children saying they trust vaccination. However, a significant portion reported hesitancy, with unfavorable perceptions about adverse reactions to vaccines, especially in lower strata. Among the reasons for not fully vaccinating children (approximately 3% of the total), parents or guardians mainly cited: the COVID-19 pandemic; fear of adverse reactions; misinformation and mistaken beliefs, such as the idea that a given disease “no longer exists”; contraindication by health professionals; and the opinion of friends and family members.

Approximately 7% of the parents or guardians interviewed reported difficulties in accessing vaccination services, citing factors such as the distance between health centers and their home or place of work, lack of time, vaccination schedules incompatible with their routine, and lack of transport or money to take their children to be vaccinated. Even with these barriers, among those who did manage to get to services, around 30% faced obstacles in vaccinating their child. Difficulties in this sense included lack of vaccines, vaccination room closed, health professionals advising against multiple vaccination on the same day, absence of staff, or the fact that it was not the day designated for that vaccine, among other problems.

The challenges encountered in vaccine hesitancy are many and of a diverse nature in Brazil, representing a multifactorial phenomenon, including misinformation, fear of adverse reactions and access difficulties as some of the main reasons, which requires diverse and multifaceted actions to resolve the issues indicated by the national survey. 

In summary, despite the challenges faced, Brazil has a solid track record of vaccination success, and the population, for the most part, demonstrates adherence and engagement in the public health actions promoted by the PNI. Since 2023, several initiatives have been implemented to overcome current difficulties, including:^4,5^

- creation of the National Pro-Vaccination Movement, an ongoing mobilization campaign to raise awareness about the importance of immunization, combat misinformation and promote confidence in vaccines through educational campaigns and involvement of local leaders;
- adoption of microplanning, a decentralized management strategy that enables identification of areas with low vaccination coverage and planning of specific actions, such as itinerant campaigns and vaccinations at strategic times and places, in order to expand access;
- modernization and expansion of the cold chain and the medical-industrial complex, ensuring adequate storage and transportation of vaccines and strengthening the national production of immunobiologicals, with incorporation of new technologies and partnerships with scientific institutions;
- enhancement of the National Health Data Network, which enables real-time monitoring of vaccination coverage and integrates information systems, facilitating data-based decision-making and monitoring of each citizen’s vaccination status;
- promoting pharmacovigilance of vaccines and other immunobiologicals, which ensures continuous monitoring of the safety of vaccines administered and enables careful assessment of benefit and risk, strengthening public confidence in the immunization program;
- implementation of the Health with Science program, aimed at disseminating scientific knowledge in a way that is accessible to the population, aiming to combat denialism and fake news, in addition to promoting greater engagement with evidence-based health practices; and
- strengthening surveillance of vaccine-preventable diseases, through improvement of notification and epidemiological investigation systems, ensuring rapid and effective responses to prevent outbreaks and reintroduction of diseases that had been eliminated.

The articles presented in this special edition of *Epidemiologia e Serviços de Saúde: revista do SUS* highlight the role of science in guiding these public policies, revisiting different aspects of vaccination coverage, challenging sociocultural and economic differences, and also presenting recommendations for Brazil to advance in this field and regain high vaccination coverage, protecting the population against the reintroduction and spread of vaccine-preventable diseases. These results provide up-to-date and scientific information on a topic of immediate relevance to the PNI and to Brazilian society as a whole.

As such, we invite readers to reflect on the studies presented in this publication and to become aware of their fundamental role in promoting vaccination, whether as health professionals, health service managers, researchers, students or citizens engaged in building a healthier society.
