# Supplementary material for: Vaccination coverage and hesitancy in Brazil: survey reveals reality and offers inputs for the National Immunization Policy
Source: Epidemiol Serv Saude. 2025 Jan 10;33(spe2):e2024638. doi: 10.1590/S2237-96222024v33e2024638.especial2.en (PMC11734604; doi:10.1590/S2237-96222024v33e2024638.especial2.en)
Supplement: Translation

# Translation: Cobertura e hesitação vacinal no Brasil: inquérito revela a realidade e oferece subsídios para a Política Nacional de Imunizações

**DOI:** 10.1590/S2237-96222024v33e2024638.especial2.pt

**Authors:** Eder Gatti Fernandes, Jadher Percio, Ethel Leonor Noia Maciel

**Affiliations:** 1 Ministério da Saúde, Secretaria de Vigilância em Saúde e Ambiente, Brasília, DF, Brasil

## INTRODUÇÃO

As coberturas vacinais no Brasil cresceram significativamente com a implementação do Sistema Único de Saúde (SUS) na década de 1990, ultrapassando 95% para diversas vacinas e contribuindo para o controle e a eliminação de várias doenças imunopreveníveis.^1^ No entanto, o Programa Nacional de Imunizações (PNI), em funcionamento desde 1973, enfrenta obstáculos, como a hesitação vacinal, impulsionada pela desinformação, pela baixa percepção do risco e pelas barreiras geográficas e administrativas no acesso aos serviços de vacinação.^2^

Entre 2020 e 2021, uma pesquisa nacional, coordenada pela Faculdade de Ciências Médicas da Santa Casa de São Paulo, foi realizada em todas as 26 capitais e no Distrito Federal, em parceria com o PNI.^3^ O estudo investigou as coberturas vacinais de crianças nascidas entre 2017 e 2018, avaliou o acesso aos serviços de vacinação, comparou dados das cadernetas de vacinação com registros administrativos e identificou as causas da hesitação vacinal. Com uma amostra de 37.836 crianças, estratificada por nível socioeconômico e ponderada por pesos amostrais, a pesquisa enfrentou desafios durante a pandemia de covid-19, especialmente devido às restrições de distanciamento físico impostas pelos municípios.

Os resultados desse inquérito ajudam a entender os desafios atuais enfrentados pelo PNI. Foi evidenciado que nenhuma vacina prevista no Calendário Nacional de Vacinação para crianças de até 2 anos atingiu 95% de cobertura vacinal, sendo que a maior parte dos parâmetros estimados esteve entre 80% e 90%. Em várias capitais, mesmo em estratos socioeconômicos mais altos, a cobertura não alcançou as metas, destacando desigualdades regionais e sociais. Aproximadamente 30% das crianças receberam pelo menos uma vacina em serviços privados, sendo essa proporção maior entre famílias no estrato mais elevado (58,9%), quando comparado à posição socioeconômica mais baixa (6,1%).

A confiança nas vacinas do serviço público foi alta em todos os estratos, com mais de 90% dos responsáveis afirmando confiar na vacinação. Contudo, uma parcela significativa relatou hesitação, com percepções desfavoráveis sobre as reações adversas das vacinas, sobretudo em estratos mais baixos. Entre os motivos para não vacinação completa das crianças (aproximadamente 3% do total), os pais ou responsáveis alegaram, principalmente: a pandemia de covid-19; o medo de reações adversas; desinformação e crenças equivocadas, como a ideia de que a doença “não existe mais”; a contraindicação de profissionais de saúde; e a opinião dos amigos e familiares.

Aproximadamente 7% dos pais ou responsáveis entrevistados relataram dificuldades para acessar os serviços de vacinação, mencionando fatores como a distância dos postos de saúde em relação à residência ou local de trabalho, falta de tempo, horários incompatíveis com a rotina, além da ausência de transporte ou recursos financeiros para levar os filhos. Mesmo com essas barreiras, entre aqueles que conseguiram chegar aos serviços, cerca de 30% enfrentaram obstáculos para vacinar a criança. As dificuldades incluíram falta de vacinas, fechamento da sala de vacinação, recomendação contrária por parte do profissional de saúde, ausência de pessoal, ou o fato de não ser o dia destinado para aquela vacina, entre outros problemas.

Os desafios encontrados para a hesitação vacinal são muitos e de natureza diversa no Brasil, representando um fenômeno multifatorial, incluindo-se desinformação, medo de reações adversas e dificuldades de acesso como alguns dos principais motivos, o que requer ações diversas e multifacetadas para solucionar as questões apontadas no inquérito nacional. 

Em síntese, apesar dos desafios enfrentados, o Brasil possui uma sólida trajetória de sucesso na vacinação, e a população, em grande parte, demonstra adesão e engajamento nas ações de saúde pública promovidas pelo PNI. Desde 2023, diversas iniciativas têm sido implementadas para superar as dificuldades atuais, incluindo:^4,5^

- A instituição do Movimento Nacional pela Vacinação, uma campanha de mobilização contínua para aumentar a conscientização sobre a importância da imunização, combater a desinformação e promover a confiança nas vacinas por meio de campanhas educativas e envolvimento de lideranças locais;
- a adoção do microplanejamento, uma estratégia de gestão descentralizada que permite identificar áreas com baixa cobertura vacinal e planejar ações específicas, como campanhas itinerantes e vacinação em horários e locais estratégicos, para ampliação do acesso;
- a modernização e expansão da rede de frio e do complexo industrial de saúde, garantindo o armazenamento e transporte adequado de vacinas e fortalecendo a produção nacional de imunobiológicos, com a incorporação de novas tecnologias e parcerias com instituições científicas;
- o aprimoramento da Rede Nacional de Dados em Saúde, que possibilita o monitoramento, em tempo real, das coberturas vacinais e integra os sistemas de informação, facilitando a tomada de decisão baseada em dados e o acompanhamento da situação vacinal de cada cidadão;
- a promoção da farmacovigilância de vacinas e outros imunobiológicos, que assegura o monitoramento contínuo da segurança das vacinas aplicadas e permite uma avaliação criteriosa de benefício e risco, reforçando a confiança pública no programa de imunização;
- a implantação do programa Saúde com Ciência, voltado para a disseminação de conhecimento científico de forma acessível à população, visando combater o negacionismo e as *fake news*, além de promover um maior engajamento com as práticas de saúde baseadas em evidências; e
- o fortalecimento da vigilância de doenças imunopreveníveis, por meio do aprimoramento dos sistemas de notificação e investigação epidemiológica, assegurando respostas rápidas e eficazes para prevenir surtos e reintrodução de doenças já eliminadas.

Os artigos apresentados neste número especial da *Epidemiologia e Serviços de Saúde: revista do SUS* (RESS) destacam o papel da ciência para orientar essas políticas públicas, revisitando diferentes aspectos da cobertura vacinal, diferenças socioculturais e econômicas desafiadoras e, ainda, apresentando recomendações para o Brasil avançar nesse campo e reconquistar elevadas coberturas vacinais, protegendo a população contra a reintrodução e disseminação de doenças imunopreveníveis. Esses resultados evidenciam informações atualizadas e científicas sobre um tema de relevância imediata para o PNI e toda a sociedade brasileira.

Assim, convidamos os leitores a refletirem sobre os estudos apresentados nesta publicação e a se conscientizarem sobre seu papel fundamental na promoção da vacinação, seja como profissionais de saúde, gestores, pesquisadores, estudantes ou cidadãos engajados na construção de uma sociedade mais saudável.
